# High quantum efficiency ruthenium coordination complex photosensitizer for improved radiation-activated Photodynamic Therapy

**DOI:** 10.3389/fonc.2023.1244709

**Published:** 2023-08-28

**Authors:** Abul Kalam Azad, Lothar Lilge, Nawaid H. Usmani, John D. Lewis, Houston D. Cole, Colin G. Cameron, Sherri A. McFarland, Deepak Dinakaran, Ronald B. Moore

**Affiliations:** ^1^ Department of Oncology, University of Alberta, Edmonton, AB, Canada; ^2^ Princess Margaret Cancer Centre, University Health Network, Toronto, ON, Canada; ^3^ Department of Chemistry and Biochemistry, The University of Texas at Arlington, Arlington, TX, United States; ^4^ Radiation Oncology Branch, National Cancer Institute, National Institute of Health, Bethesda, MD, United States; ^5^ Department of Surgery, University of Alberta, Edmonton, AB, Canada

**Keywords:** radioPDT, radiodynamic therapy, PDT - photodynamic therapy, radiation, PLGA (poly-lactic-co-glycolic acid), ruthenium

## Abstract

Traditional external light-based Photodynamic Therapy (PDT)’s application is limited to the surface and minimal thickness tumors because of the inefficiency of light in penetrating deep-seated tumors. To address this, the emerging field of radiation-activated PDT (radioPDT) uses X-rays to trigger photosensitizer-containing nanoparticles (NPs). A key consideration in radioPDT is the energy transfer efficiency from X-rays to the photosensitizer for ultimately generating the phototoxic reactive oxygen species (ROS). In this study, we developed a new variant of pegylated poly-lactic-co-glycolic (PEG-PLGA) encapsulated nanoscintillators (NSCs) along with a new, highly efficient ruthenium-based photosensitizer (Ru/radioPDT). Characterization of this NP via transmission electron microscopy, dynamic light scattering, UV-Vis spectroscopy, and inductively coupled plasma mass-spectroscopy showed an NP size of 120 nm, polydispersity index (PDI) of less than 0.25, high NSCs loading efficiency over 90% and *in vitro* accumulation within the cytosolic structure of endoplasmic reticulum and lysosome. The therapeutic efficacy of Ru/radioPDT was determined using PC3 cell viability and clonogenic assays. Ru/radioPDT exhibited minimal cell toxicity until activated by radiation to induce significant cancer cell kill over radiation alone. Compared to protoporphyrin IX-mediated radioPDT (PPIX/radioPDT), Ru/radioPDT showed higher capacity for singlet oxygen generation, maintaining a comparable cytotoxic effect on PC3 cells.

## Introduction

Cancer is a leading cause of death worldwide, second to cardiovascular diseases. In 2020, an estimated 18.1 million cases of cancer and nearly 10 million deaths were reported globally (1). Conventional cancer treatments such as chemo- and radiotherapies have clinically been proven as effective therapeutics for many cancer types; however, clinical scenarios with radiation or chemo-resistant cancers still exist where the therapeutic effect is not durable and leads to treatment-refractory disease over time (2, 3). Alternative cancer treatment modalities have been developed, including photodynamic therapy (PDT), given its high selectivity and non or minimal invasiveness (4, 5). In PDT, the photosensitizer (PS) is activated by light irradiation that generates reactive oxygen species (ROS), mediating the cell-killing mechanism. However, the tissue penetration depth of light in non-invasive clinical PDT systems is often less than 1 cm, thus limiting the application of PDT to tumors that are located superficially on the skin and endoscopically accessible subcutaneous tissues (5, 6). Deeper situate or thicker tumors can still be treated with PDT, but invasive interstitial light catheters are required to deliver the activating light effectively (7). In recent years, radiation-activated PDT (radioPDT), where the photosensitizer uses energy from X-ray photons rather than optical photons for activation, is gaining momentum to mitigate the limitation of visible/near-infrared (NIR) light sources in penetrating deep tissue structures (7, 8). X-ray photons have a much larger penetration depth and can be used to induce radioluminescence within nanoscintillators (NSCs), which in turn will activate the adjacent PSs via Forster resonance energy transfer (FRET) to generate ROS (9).

In PDT, finding an appropriate PS is challenging. For clinical application, the ideal PS factors to be considered include chemical purity, stability in physiologic conditions, low dark toxicity, higher tumor selectivity, localization to critical intracellular structure to trigger cell death, quick clearance from the body, and high ROS generation under activating light for efficient therapeutic yield (10). The latter is important, particularly with low-energy input fluence treatments (11). Protoporphyrin IX (PPIX) is one of the most commonly studied PSs in PDT. PPIX and its prodrug 5-aminolevulinic acid (5-ALA), a naturally occurring amino acid, are precursors in the biosynthetic heme pathway. Upregulation of this pathway leads to PPIX accumulating in tumors, which is favorable for treating tumors with PDT (12–15). Nevertheless, the clinical application of PPIX, 5-ALA, and other porphyrin derivatives like Photofrin in PDT remain limited to superficial cancers, including non-melanoma skin cancers, bladder, esophageal, lung and head and neck cancers (5, 16, 17).

In contrast, radiotherapy is widely used to treat approximately 50% of all cancer patients (18). Modern radiotherapy can be highly focused anywhere in the body with intensity-modulated radiotherapy and image-guided radiotherapy (2). By doing this, the toxicity to efficacy ratio of radiotherapy has been greatly optimized. Nevertheless, the short and long-term toxicities of radiotherapy is a major limiting factor to expanding its application in cancer care, and causes significant permeant side-effects to patients (19).

**Figure 1:**
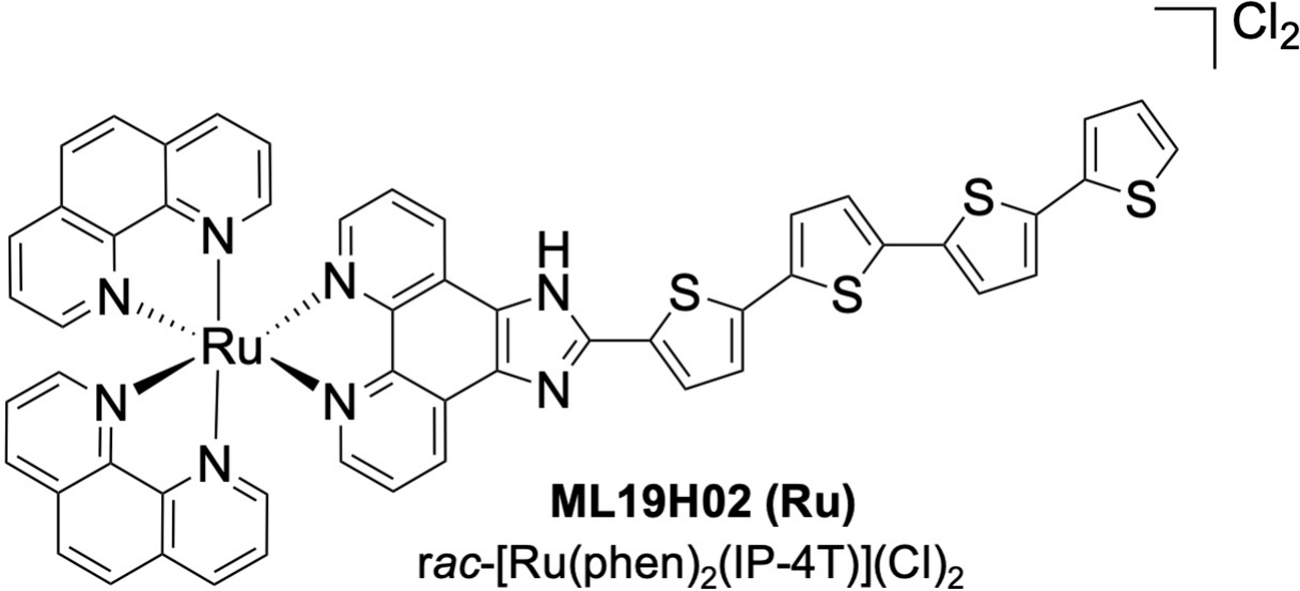
Ruthenium coordination complex photosensitizer, ML19H02.

To address this, a new field has emerged at the crossroads between radiation and PDT, where radiation energy excites the PS for subsequent photochemical activity in a phenomenon termed radiation-activated PDT (radioPDT). Doing so allows the therapeutic effect of radiotherapy to be augmented by radioPDT without additional radiation dose, which can lead to additional radiotoxicity. Achieving radioPDT through nanoparticles also allows for the opportunity for multimodal strategies such as therapy and diagnostic (theranostic) agents (20, 21). We previously demonstrated one such radioPDT agent consisting of polyethylene glycol conjugated to poly-lactic-co-glycolic acid (PEG-PLGA) encapsulating LaF_3_:Ce^3+^ NSC and PPIX PS nanoparticle (PPIX/radioPDT NP), with an impressive performance *in vitro* and *in vivo* (9). Given the emergence of ruthenium (Ru) coordination complexes as PSs with attractive properties for PDT, particularly with much higher phototherapeutic index and quantum yield (11, 22–24), we hypothesized a more efficient radioPDT system could be generated by substituting PPIX for a Ru PS (**Figure 1**). Herein, we tested ML19H02 (Ru) within our radioPDT NP (Ru/radioPDT) construct *in vitro* using the PC3 cell line, an aggressive prostate cancer cell line derived from grade IV metastatic prostatic adenocarcinoma (25). The characteristics and effectiveness of Ru/radioPDT were evaluated by light irradiation and under X-ray irradiation and compared to the previously reported PPIX/radioPDT (9).

## Methods

PPIX (P8293) was purchased from Sigma Aldrich (Oakville, ON, CA). Prostate cancer (PC3) cell line (CRL-1435) was purchased from ATCC (Manassas, VA, USA). Singlet oxygen sensor green (SOSG) kit was purchased from ThermoFisher, MA, USA. Dulbecco’s modified Eagle’s medium (DMEM) was purchased from Life Technologies, Carlsbad, CA, USA.

### Photosensitizer synthesis

ML19H02 is the Ru PS with the chemical formula *rac*-[Ru(phen)_2_(IP-4T)](Cl)_2_, where phen=1,10-phenanthroline and IP-4T is imidazo[4,5-*f]* phenanthroline tethered to α-quaterthiophene. ML19H02 was prepared as previously described (24) and characterized by 1D and 2D ^1^H NMR spectroscopy and Electrospray Ionization (ESI^+^) mass spectrometry. Its purity was estimated at >95% by HPLC.

### Nanoparticle synthesis

The synthesis technique follows our previously reported radioPDT production in Dinakaran et al. (2020) with some modifications (9). Briefly, 45 mmol of lanthanum chloride (LaCl_3_, 99.9%) and 5 mmol of cerium (III) chloride (CeCl_3_.6H_2_O, 99.9%) were dissolved in 24 mL anhydrous methanol by stirring and vortexing at room temperature for 30 minutes. Similarly, 100 mmol of ammonium fluoride (NH_4_F) was dissolved in 180 mL of anhydrous methanol. Next, the NH_4_F solution was maintained at 70 ^0^C with constant stirring while lanthanum (III) chloride and cerium (III) chloride solution (90% LaCl_3_, 10% CeCl_3_) was added to the NH_4_F dropwise and kept stirring for 2 hrs. The reaction occurred in a nitrogen protected environment with all oxygen and water purged. The resultant LaF_3_:Ce^3+^ (10% doping ratio) NSC was pelleted by ultracentrifugation, and the final product was resuspended in water. The quality of LaF_3_:Ce^3+^ NSC was characterized by transmission electron microscopy (TEM), UV-Vis, and dynamic light scattering (DLS).

Next, as described earlier, LaF_3_:Ce^3+^ NSC and PPIX or Ru were encapsulated into PEG-PLGA by the nanoprecipitation method into PPIX/radioPDT NP or Ru/radioPDT, respectively (9). For PPIX/radioPDT NP, 100 mg of PEG-PLGA, 15 mg of LaF_3_:Ce^3+^ NSC, and 2 mg of PPIX were dissolved in 10 mL of acetonitrile by sonicating for 15 mins and rotating at room temperature for 30 mins. For Ru/radioPDT, 2 mg Ru, 100 mg PEG-PLGA, and 15 mg/mL LaF_3_:Ce^3+^ NSC were quickly dissolved in 10 mL of acetonitrile without sonication. Of note, the PS and NSC loading amounts were chosen based on the favorable physical properties of the resultant nanoparticle. The organic phase reactants were added dropwise to 200 mL of MilliQ water using a variable flow peristaltic pump. The organic solvent was removed by vacuum evaporation using a rotavapor system, and the left-over solution was stirred at room temperature overnight. Next, the NPs were purified and concentrated using a tangential flow filtration (TFF) to generate PPIX/radioPDT NP or Ru/radioPDT NP. A hollow fiber cartridge (GE Healthcare, MA, USA) with 500,000 NMWC pore size was used for TFF. Via TFF, the radioPDT NP was washed in 20X excess milliQ H_2_O with a transmembrane pressure maintained at 1 bar. A final volume of 3 mL of NPs was collected, and the particle recovery and encapsulation were measured by DLS and TEM.

### UV-Vis spectrometry and dynamic light scattering (DLS), zeta potential

UV-Vis absorbance spectra were measured to ensure the successful loading of PS in PEG-PLGA. 1.5 µl of diluted radioPDT NP was placed in a nanodrop (DS-11 FX^+^, DeNovix Inc., Wilmington, DE, USA), and the absorbance spectra were collected over a range of 190-800 nm. Dynamic Light Scattering (DLS) was used to determine the size distribution, particle concentration, and polydispersity index (PDI) of the NPs. The sample was diluted 1000X in milliQ H_2_O, and 1 mL of solution was taken in a cuvette for measurement using Brookhaven Zetasizer (Brookhaven, Holtsville, NY, USA). The zeta potential was also measured with the particles suspended in milliQ H_2_O.

### Transmission electron microscopy (TEM)

A 10x dilution in milliQ H_2_O (3 µL) was placed on a 400-mesh copper grid coated with carbon (Electron Microscopy Sciences, PA, USA), allowing 3 minutes to stabilize before excess water was removed with a filter paper. 3 µl of 2% uranyl acetate (UA) was added to counterstain the grid for 2 minutes before the excess was similarly removed. The NPs were imaged on a JEM 2100 TEM (JEOL, Tokyo, Japan) using a beam energy of 200 kV under a Gatan Quantum GIF energy imaging filter. Images were captured with a Orius SC200D camera under brightfield conditions.

### NP stability assay

The stability of the NPs was determined by size, zeta potential and UV-Vis measurements in physiologic media. The NPs with different constituents (PEG-PLGA, NSC/PEG-PLGA, PPIX/radioPDT, and Ru/radioPDT) were added to 8% FBS containing phenol red-free DMEM medium. Samples were incubated at 37°C for 24 hrs and 48 hrs. The size distribution and zeta potential of the NPs were measured using Brookhaven Zetasizer (Brookhaven, Holtsville, NY, USA). UV-Vis was measured using a nanodrop (DS-11 FX^+^, DeNovix Inc., Wilmington, DE, USA).

### Singlet oxygen yield measurement

Single oxygen yield was measured via the SOSG probe and its direct fluorescence similar to Dinakaran et al. (9). For the SOSG measurement, 1×10^12/^mL of radioPDT NP and control NSC NPs, and UV-Vis standardized equivalent PPIX or Ru were added to a 96-white well plate. Subsequently, 10 μM of SOSG was added, and the volume was adjusted to 100 μL with 2X PBS at a pH of 7.4. One plate was kept in the dark while the other was irradiated at 5 J/cm^2^ with a 402 nm monochromatic LED light source (HouLight, China) at a fluence of 12 mW/cm^2^, which was calibrated for fluence and homogeneity (+/- 5%) using a Thorlabs PM100D photometer (Thorlabs Inc, Newton, NJ, USA). After light irradiation, the SOSG fluorescence was measured (excitation 485 nm, emission 520 nm) by a FLUOstar Omega plate reader (BMG Labtech, Ortenberg, Germany). Each light irradiated experimental condition was normalized to the respective dark condition for analysis.

For direct singlet oxygen measurement, singlet oxygen sensitization (Φ_Δ_) was measured at room temperature as suspensions in D_2_O, or a solution in 99:1 D_2_O:DMSO for PPIX. The values were inferred from the intensity of the singlet oxygen emission band near 1276 nm, measured on a PTI Quantamaster spectrometer (Horiba Scientific, Kyoto, Japan) equipped with a near-infrared sensitive Hamamatsu R5509-42 photomultiplier tube cooled to −80 ^0^C. The most intense absorbance region in the excitation spectrum was chosen to do relative quantification. The quantum yield was calculated by relative actinometry, as shown in Equation 1, where *I* is the integrated intensity of the emission, *A* is the baseline-corrected absorbance of the solution or suspension at the excitation wavelength, and *η* is the refractive index of the solvent. The subscript *S* denotes the standard solution [Ru(bpy)_3_](PF_6_)_2_ in acetonitrile, for which Φ*_Δ,s_* =0.56 (26).


Equation 1 $$\Phi_{\text{Δ}}=\Phi_{\text{Δ},s}\left(\frac{I}{I_{s}}\right)\left(\frac{A_{s}}{A}\right)\left(\frac{\eta^{2}}{\eta_{s}^{2}}\right)$$

### *In vitro* therapeutic effect

PC3 prostate cancer cell line was cultured in DMEM with 8% FBS supplemented with 100 U/mL penicillin and 100 μg/ml streptomycin (Life Technologies, USA). Cells were cultured under standard conditions in a humidified incubator at 37°C with 5% CO_2_.

Cytotoxicity was measured as cell viability by colorimetric assay and cell survival by clonogenic assay. These assays were chosen to more closely examine PDT/radioPDT effect alone, as represented by the colorimetric assay, and radioPDT effect in combination with radiotherapy as represented by the clonogenic assay. For the colorimetric assay, PC3 cells were incubated overnight at 5000 cells/well density in black, clear bottom, 96 well plate (ThermoFisher, Waltham, MA, USA). 1×10^12^ NPs/mL and UV-Vis standardized equivalent amount of free PPIX or Ru were added to the cells for 3 hrs. For light activated PDT, cells were irradiated at 2 J/cm^2^ (12 mW/cm^2^) with a monochromatic LED. Emitting 405 nm light For radiation activated PDT (radioPDT), cells were irradiated at 8 Gy using a Cesium Irradiator (JL Shepherd and Associates, San Fernando, CA, USA). The cell media was changed 2 hrs post-irradiation to remove the nanoparticles and incubated for 72 hrs prior to viability measurement with Alamar blue dye (10% v/v) using fluorescence measurement (excitation 544 nm, emission 590 nm) with a FLUOstar Omega plate reader.

For the clonogenic cytotoxicity assay PC3 cells were seeded into 12-well plates at a density of 500 cells/well. The cells were treated as described for the colorimetric viability assay but with a lower radiation dose of 3 Gy in a single fraction to better represent the contemporary understanding of prostate radiobiology and radiotherapy dose single fraction as used clinically (27). In contrast, 8 Gy was necessary in the colorimetric assay as there was little contribution from radiotherapy’s DNA damage-induced lack of clonogenicity in the 72 hr assay period in comparison to PDT’s more immediate cytotoxic effects, necessitating a higher activating radiation dose for radioPDT to obtain a cell viability readout. Cell media was changed every 3 days until the plate was developed at day 14 by washing once with PBS, fixation with 1% formaldehyde and 1% methanol, and staining with 0.05% crystal violet. The colonies were counted by scanning the plates on a High Content MetaXpress SLS (Molecular Devices, San Jose, CA, USA) system. Plating efficiency was calculated to derive surviving fractions as previously established for clonogenic assays (28).

### Cellular uptake and organelle localization of NPs

PC3 cells were grown to 80% confluency in Aclar film in 6-well plate and then treated with Ru/radioPDT for 4 hrs. Primary fixation of cells was done in 2% PFA + 2.5% GTA/0.1 M Cacodylate buffer with 2 mM CaCl_2._ After a series of washing steps, the cells were dehydrated in increasing ethanol concentrations, followed by infiltration in 1:1 Durcupan resin and acetone. Cells were polymerized at 60°C over 48 hrs by placing the Aclar sheet on top of a beam capsule with the cells facing down. After polymerization, the Aclar sheet was peeled off from the block, cut with a Leica EM UC6 ultramicrotome, and transferred to a carbon grid for TEM imaging to detect the presence of NPs within the cells via detecting Lanthanum within the LaF_3_:Ce^3+^ NSC using elemental mapping by energy-filtered TEM (EFTEM) on a JEM 2100 TEM machine (JEOL, Tokyo, Japan) with a Gatan Quantum GIF system (Gatan, CA, USA).

### Statistics

All experiments were done in triplicate and repeated at least 3 times. Data are reported as mean ± SEM. One-way ANOVA was used to evaluate for statistically significant difference with a p-value cutoff of <0.05 using Prism 8 (GraphPad 5, San Diego, USA).

## Results

### Nanoparticle synthesis

**Figure 2:**
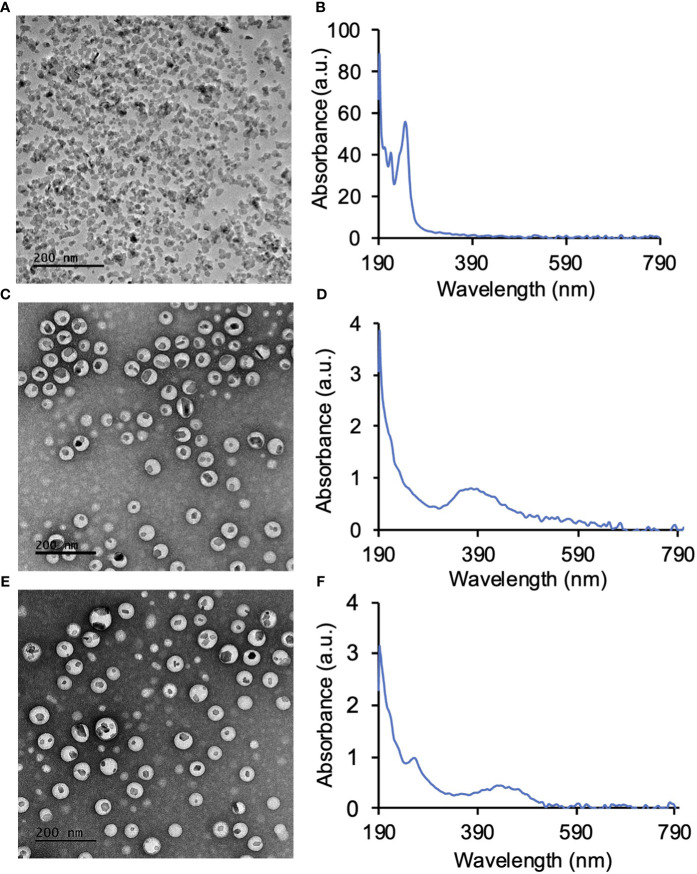
Synthesis of LaF_3_:Ce^3+^ nanoscintillators (NSC) and PEG-PLGA encapsulation of LaF_3_:Ce^3+^ NSC and photosensitizers. **(A)** TEM image of NSC and **(B)** the corresponding UV-Vis absorption spectroscopy of NSC. **(C)** PEG-PLGA encapsulated protoporphyrin IX (PPIX) and NSC (PPIX/radioPDT). **(D)** UV-Vis absorption spectrum of PPIX/radioPDT. **(E)** PEG-PLGA encapsulated Ruthenium (Ru) and LaF_3_:Ce^3+^ NSC (Ru/radioPDT). **(F)** UV-Vis absorption spectrum of Ru/radioPDT.

LaF_3_:Ce^3+^ NSC at a 10% dopant ratio (**Table 1**) was synthesized in a single step wet chemistry process (**Supplemental Flowchart 1**) as previously reported (9). TEM of LaF_3_:Ce^3+^ NSC revealed hexagonal nanocrystal structures (**Figure 2A**), and the dynamic light scattering (DLS) measurement showed a homogenous population (PDI <0.2) of LaF_3_:Ce^3+^ NSC with an average size ranging from 30 ± 7 nm. UV-Vis absorption spectroscopy is shown in **Figure 2B**.

**Table 1:** ICP-MS of Lanthanum and Cerium in radioPDT NPs.

Radio/POT NPs	Lanthanum (mg/ml)	Cerium (mg/ml)
PPIX/radioPDT	3.94	0.87
Ru/radioPDT	3.76	1.0

LaF_3_:Ce^3+^ NSC and PS (PPIX and Ru) were then encapsulated into polymeric PEG-PLGA NP (**Supplemental Flowchart 2**). TEM showed the LaF_3_:Ce^3+^ NSC encapsulated within the PEG-PLGA NP to produce the Ru/radioPDT and PPIX/radioPDT systems (**Figures 2C, E**). UV-Vis measurements showed the presence of PPIX or Ru in PPIX/radioPDT or Ru/radioPDT, respectively (**Figures 2D, F**). Together, these data showed the successful loading of LaF_3_:Ce^3+^ NSC and photosensitizers into PEG-PLGA.

### NP physical characteristics

**Figure 3:**
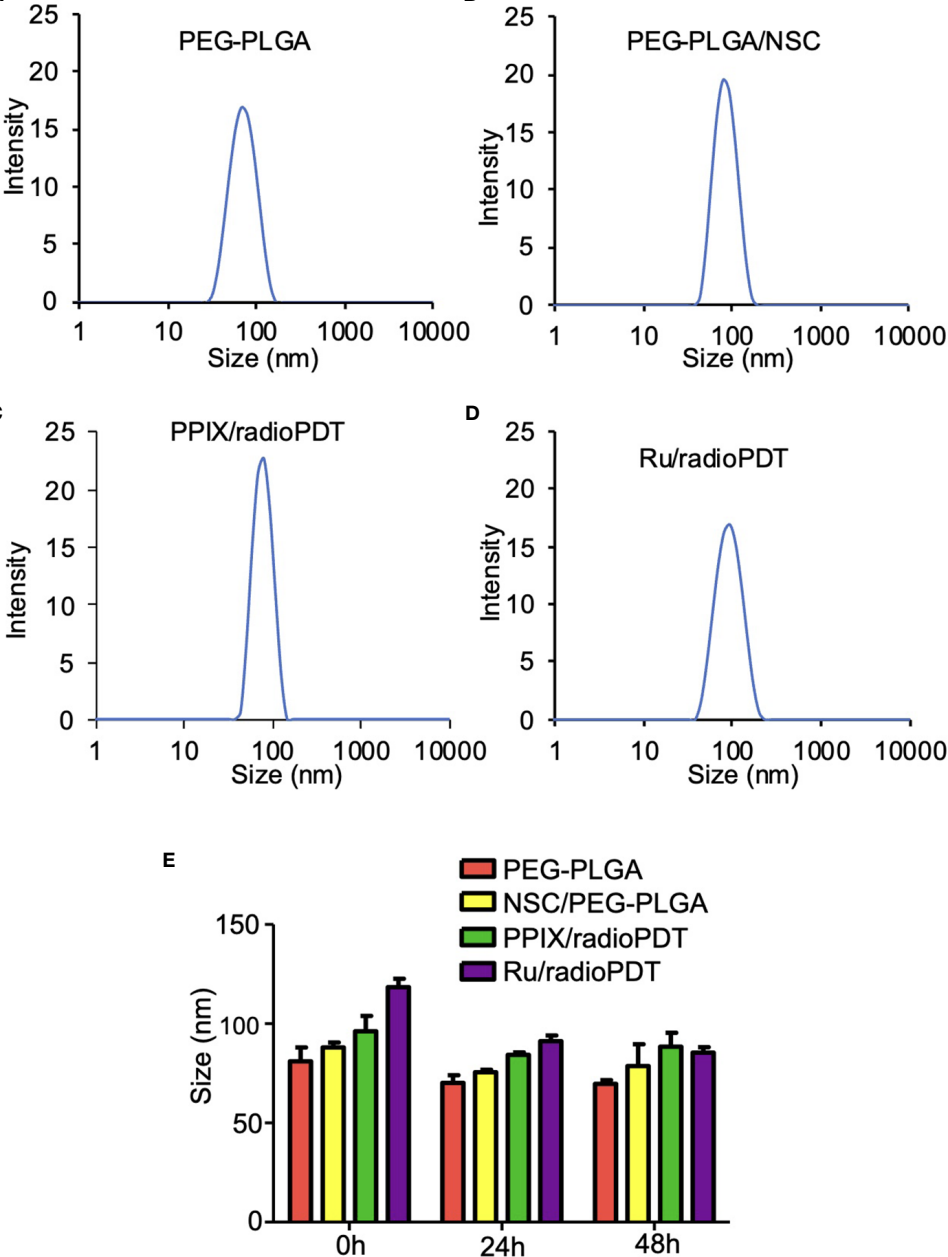
Size and stability of the nanoparticles (NPs) and radioPDTs. **(A)** The size distribution of PEG-PLGA NP as measured by Dynamic light scattering (DLS). **(B)** The DLS of LaF_3_:Ce^3+^ NSC loaded into PEG-PLGA, **(C)** PPIX/radioPDT, and **(D)** Ru/radioPDT. **(E)** The size distribution of PEG-PLGA NP, PEG-PLGA/NSC NP, PPIX/radioPDT, and Ru/radioPDT for the indicated time as measured by DLS. Mean ± SEM; n = 3.

DLS measurement showed the average hydrodynamic diameter of the unloaded PEG-PLGA NP was 81 ± 5 nm, while the sizes of PEG-PLGA loaded with LaF_3_:Ce^3+^ NSC was 88 ± 2 nm (**Figures 3A, B**). The full radioPDT system consisting of PEG-PLGA loaded with LaF_3_:Ce^3+^ NSC and PPIX (PPIX/radioPDT) was 96 ± 6 nm and 118 ± 3 nm for the LaF_3_:Ce^3+^ NSC, and Ru loaded PEG-PLGA (Ru/radioPDT) (**Figures 3C, D**). The PDI of all the NPs preparation was less than 0.25, indicating a relatively homogenous population of NPs. The measured average zeta potential was -17.4 ± 0.7 mV for Ru/radioPDT NP, as opposed to -27.4 ± 0.5 mV and −19 ± 1 mV for PPIX/radioPDT NP and NSC NP, respectively.

After radioPDT synthesis, we sought to determine its stability in physiologic media for determining the effective treatment time window. DMEM (without phenol red) supplemented with 8% FBS at 37°C was used to incubate the NP and the relevant controls over 48 hrs. The size and PDI measured by DLS showed the NPs after 24 hrs and 48 hrs remained relatively unchanged over these time points (**Figure 3E**), suggesting the NPs maintain their stability without aggregation or breakdown in physiologic serum conditions. In addition, the zeta potential (**Supplemental Table 1**) and UV-Vis (**Supplemental Figure S2**) measurements showed stable negative charges and the presence of photosensitizer for a period of 48 hours under physiological conditions. Taken together, these data confirmed the stability of radioPDT NPs under these conditions.

### Singlet oxygen generation under 405 nm light irradiation

**Figure 4:**
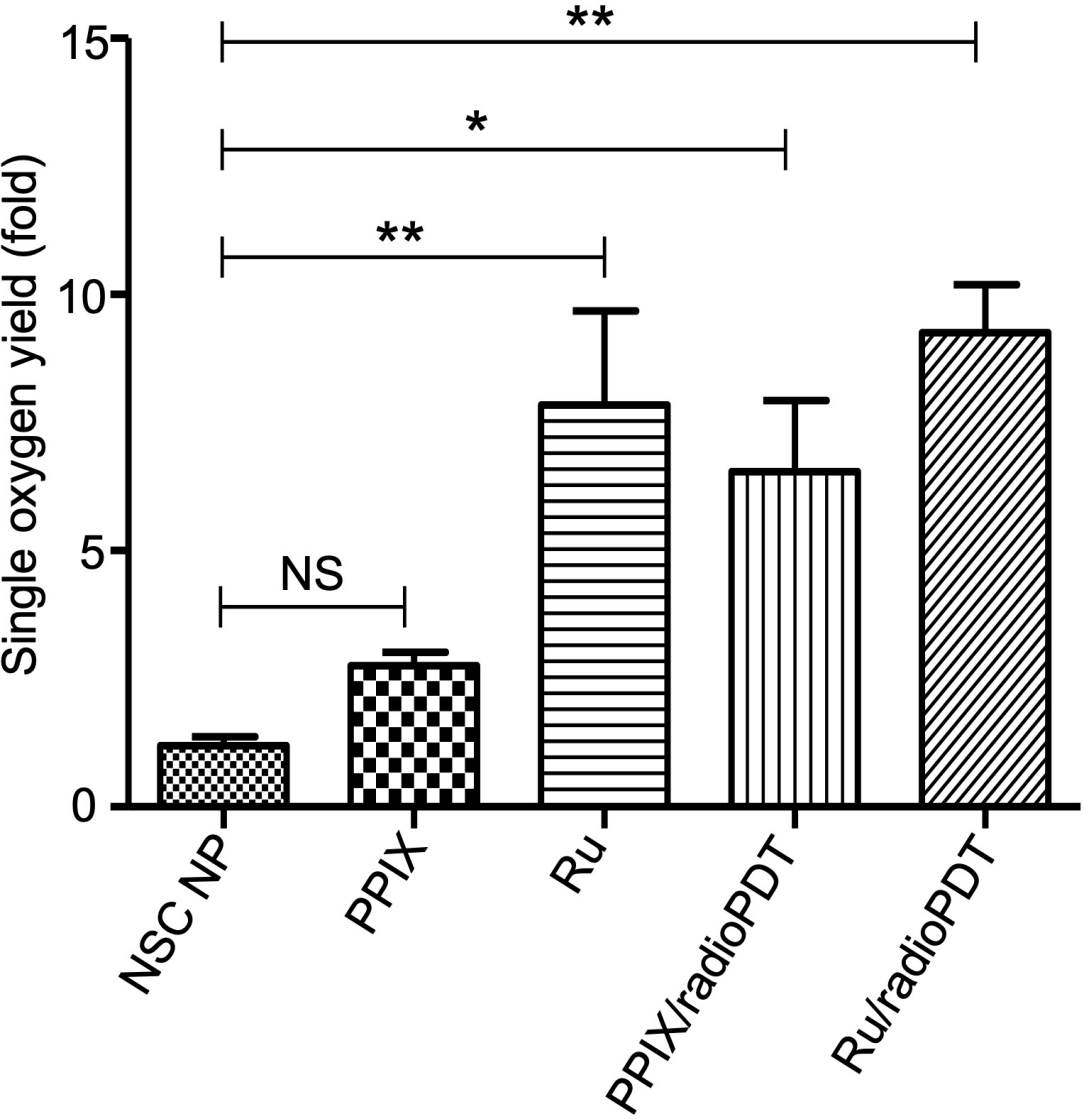
Generation of singlet oxygen by radioPDTs. Generation of singlet oxygen in NSC NP, PPIX, Ru, PPIX/radioPDT and Ru/radioPDT as measured by the SOSG probe after 5 J/cm2 radiant exposure (irradiance 12 mW/cm2) with monochromatic light. Each condition was normalized to its respective non-irradiated (dark) condition. Mean ± SEM; n = 5; *P < 0.05, **P < 0.01 by one-way ANOVA. NS, not significant.

In PDT, the cytotoxic effect relies on the ability of PS to generate ROS, importantly singlet oxygen (10, 29). To determine the efficiency of Ru/radioPDT, we used the singlet oxygen sensor green (SOSG) kit (ThermoFisher, MA, USA) as a highly specific probe for singlet oxygen. The generation of singlet oxygen, as measured by SOSG, was significantly higher in free Ru compared to PPIX, as would be expected by Ru’s significantly higher quantum yield (30) (**Figure 4**). SOSG signal for free Ru was higher than free PPIX and it was also higher for Ru/radioPDT NP compared to PPIX/radioPDT NP. Both PPIX/radioPDT NP and Ru/radioPDT NP gave significantly higher SOSG signals compared to control NSC NPs without PS (**Figure 4**). Of note, the average concentration of free PS and nanoparticulated PS was standardized, indicating any difference in efficacy may be due to absorption, triplet state formation, and/or sensitization pathways. These trends were corroborated by singlet oxygen quantum yield measurements performed in D_2_O based on singlet oxygen emission, whereby Ru/radioPDT NP produced a singlet oxygen quantum yield of up to 5% compared to well under 1% for PPIX/radioPDT and free PPIX or NSC NP.

### Cytotoxicity of Ru/radioPDT NP compared to PPIX/radioPDT NP

**Figure 5:**
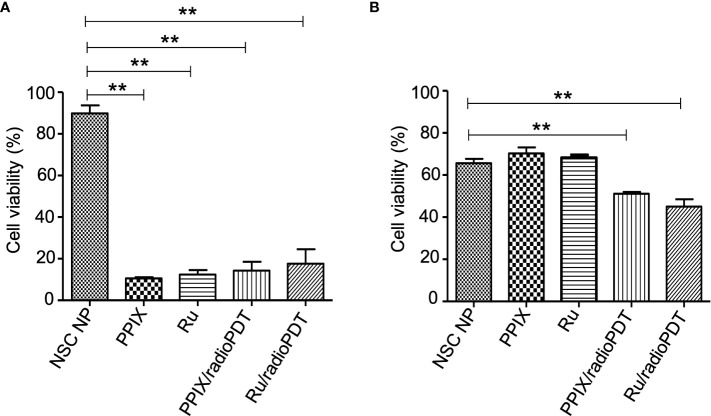
Cytotoxic effect of radioPDT NPs on PC3 cell viability. **(A)** PC3 cells were treated with radioPDT NPs and an equivalent amount of control reagents and then irradiated with 2 J/cm^2^ (12 mW/cm^2^) 402-nm blue light. Each condition was normalized to its respective non-irradiated condition. Mean ± SEM; n = 4; **P < 0.01 by one-way ANOVA. **(B)** Cells treated under similar conditions but irradiated with 8 Gy single dose radiation instead of light. Each condition was normalized to its respective non-irradiated condition. Mean ± SEM; n = 4; **P < 0.01 by one-way ANOVA.

Next, we sought to determine the photodynamic efficacy of Ru/radioPDT compared to previously reported PPIX/radioPDT. For comparison, PC3 cells were treated with Ru/radioPDT NP or PPIX/radioPDT NP (and the analogous light-based PDT conditions) and assessed for cell viability using the colorimetric assay. Both PPIX and Ru and their NSC formulations exhibited similar cytotoxicity when irradiated with 402-nm light at a radiant exposure of 2 J/cm^2^ (irradiance of 12 mW/cm^2^) (**Figure 5A**). The NSC NPs alone did not produce any photocytotoxicity under similar conditions. Following a single dose ionizing radiation of 8 Gy, free PPIX or Ru did not produce any measurable cytotoxic effects (**Figure 5B**). Ru/radioPDT NP was more potent than PPIX/radioPDT NP but not statistically different.

**Figure 6:**
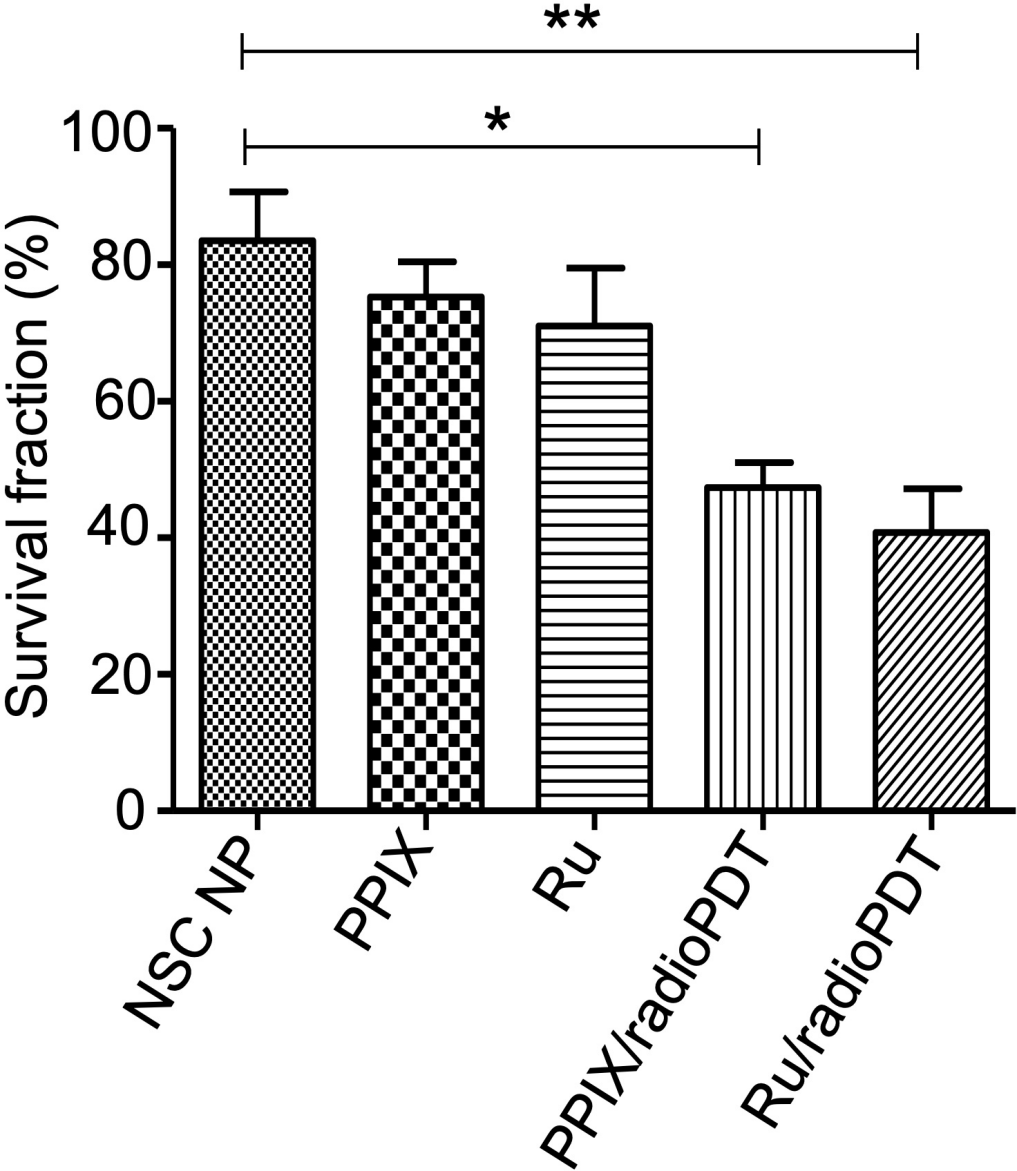
Colony forming ability of PC3 cells treated with radioPDT NPs and radiation. PC3 cells were treated with radioPDT NPs and an equivalent amount of control reagents and then irradiated with 3 Gy single dose radiation. Mean ± SEM; n = 4; *P < 0.05 by one-way ANOVA; **P < 0.01 by one-way ANOVA.

Further investigations examined the combined effect of radioPDT and radiotherapy on PC3 survival following delivery of a single 3 Gy ionizing radiation dose in combination with Ru/radioPDT NP or PPIX/radioPDT NP. PC3 survival was significantly decreased in both Ru/radioPDT and PPIX/radioPDT compared to radiation alone, with cell survival reduction of 49% and 57% of the radiation-only condition, respectively (**Figure 6**, **Supplemental Figure S3**). A statistically nonsignificant trend toward decreased survival in the Ru/radioPDT NP group was seen over PPIX/radioPDT NP at this dose of radiation.

### Cellular uptake and cytoplasmic localization of radioPDT NPs

**Figure 7:**
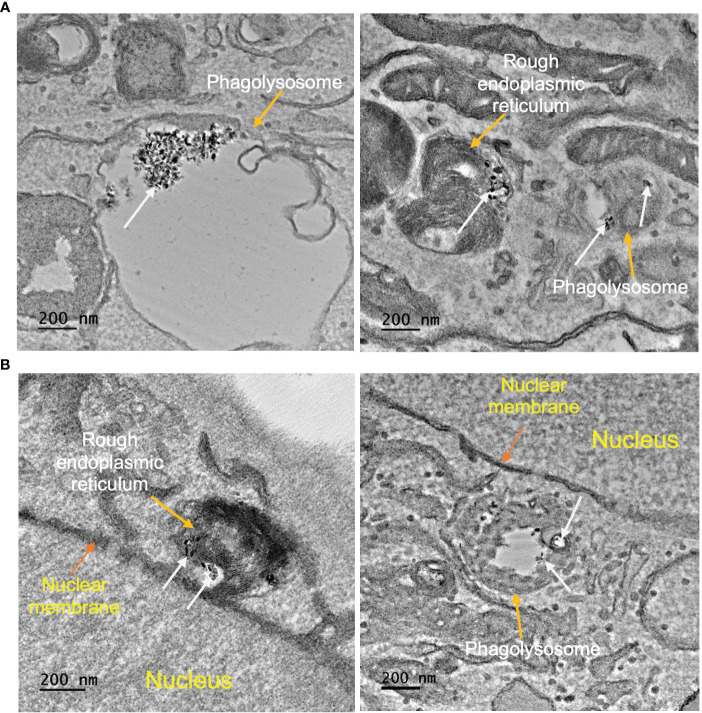
Ru/radioPDT uptaken by PC3 cells. **(A)** Representative TEM images of LaF_3_:Ce^3+^ NSC (white arrow) taken up by PC3 cells; left panel shows LaF_3_:Ce^3+^ NSC inside the lysosome, and the right panel shows LaF_3_:Ce^3+^ NSC inside endoplasmic reticulum. **(B)** Representative TEM images showing LaF_3_:Ce^3+^ NSC remains outside the nucleus. Scale bar 200 nm.

**Figure 8:**
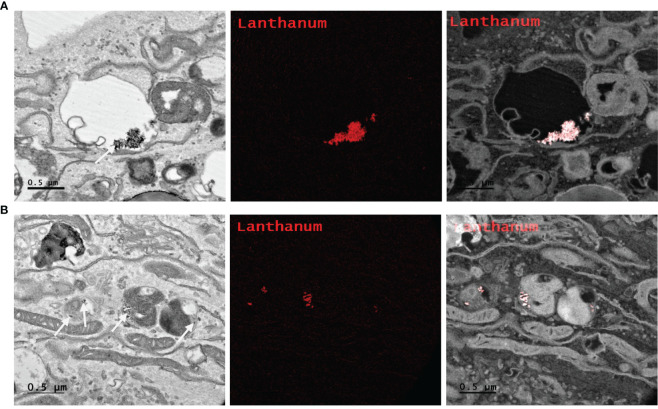
Elemental mapping of Lanthanum by Energy filtered TEM (EFTEM). Representative images of different parts of the PC3 cell **(A, B** left**)** with EFTEM to identify the presence of Lanthanum (left, white arrow; middle, red; right, merge) as a Ru/radioPDT NP constituent. Scale bar 0.5 µm.

Determining intracellular localization of Ru/radioPDT NP indicates the likely organelles affected by the short-range singlet oxygen species during PDT effect in the context of our experimental setup. PC3 cells treated with Ru/radioPDT NP were prepared for TEM imaging as described in the method section. Of note, acetone was used as the fixative during the sample preparation, which did not significantly interfere with the stability of Ru/radioPDT NP. TEM demonstrates the localization of the high contrast inorganic LaF_3_:Ce^3+^ NSC in cytoplasmic organelles, including in phagolysosomes and endoplasmic reticulum (**Figure 7A**), but remaining outside the nucleus (**Figure 7B**). This is further confirmed by elemental mapping of Lanthanum EFTEM (**Figures 8A, B**). Together, Ru/radioPDT NP appears to be endocytosed into PC3 cells and localizes to organelles outside the nucleus, particularly critical structures such as the endoplasmic reticulum, and presumably damages these structures when the PDT effect is induced by radiation to achieve cell kill. This contrasts with the well-known DNA damage-mediated radiotherapy effect (31).

## Discussion

RadioPDT is an emerging field in anticancer therapy for the non-invasive treatment of deep-seated tumors, otherwise not amenable to light-dependent PDT. The X-ray absorption and luminescence produced by the LaF_3_:Ce^3+^ NSC upon irradiation can, in turn, activate PSs to generate ROS. The level of activation and efficacy is far in excess of what is achievable by several organic PSs, such as PPIX derivatives under X-ray radiation (7, 9, 32). Incorporating an NSC with PPIX into a nanoparticle construct can significantly improve the radioPDT process. This has been reported in multiple other previous studies with similar nanocarriers (9, 33, 34). However, PPIX’s relatively low quantum yield and high photobleaching rate may still limit the overall system’s efficacy (9). Here, a new NP complex consisting of 3 components: polymer PEG-PLGA as a carrier for drug delivery, LaF_3_:Ce^3+^ NSC for X-ray energy capture and transfer to a Ru coordination complex PS demonstrates a potentially more efficient method of conducting radioPDT. This new Ru/radioPDT NP exhibits several favorable characteristics over PPIX/radioPDT NP. The synthesized Ru/radioPDT NP was comparable to previously reported PPIX/radioPDT with a uniform size distribution of 100-120 nm. This size range corresponds to the ideal size required for increasing the circulatory half-life and the subsequent bioavailability of NPs at the tumor site (35, 36). Ru/radioPDT NP also exhibited a moderately charged -17.4 mV zeta potential, as opposed to -27 mV for PPIX/radioPDT NP, which helps maintain stability in aqueous conditions and in circulation (37), but the more positive charge predicts for increased likelihood of Ru/radioPDT NP to interact with negatively charged cell membranes and be endocytosed and localize into critical organelle structures such as the endoplasmic reticulum (38). In addition, the measured stability of the NPs was well preserved for up to 48 hrs in physiologic media, which allows adequate circulation and cellular uptake time before it can be activated by radiation.

The singlet oxygen generation with Ru/radioPDT is higher than PPIX/radioPDT for the same light irradiation parameters. Based on this observation, one expects a higher PC3 cell killing efficiency under similar conditions, but cell death was exceedingly efficient in all the experimental conditions upon light irradiation. Radiation activation augmented the PC3 cytotoxicity in both radioPDT NP formulations, with an additional 16% improvement in efficacy for Ru/radioPDT over PPIX/radioPDT. Curiously, this did not manifest as a statistically significant difference despite better singlet oxygen yield, but this perhaps relates to the low input energy of 3 Gy in the clonogenic assay. In a multifractionated regimen, similar to clinical radiotherapy use, a statistically significant result could be likely. Mechanistically, Ru/radioPDT was taken up by the cells and entered into different cytoplasmic structures but without entering the nucleus, suggesting that the augmented effect of Ru/radioPDT in PC3 cell death, is related to the damage of cytoplasmic organelles, particularly the endoplasmic reticulum, which is known to be sensitive to ROS stress and affects cell survival (39). Further experiments are needed to understand the mechanisms of Ru/radioPDT induced cell death upon radiation activation in detail.

## Conclusion

To improve radioPDT efficiency and increase its ability to provide an additional anticancer effect over radiation alone, a new radioPDT NP system consisting of PEG-PLGA encapsulated LaF_3_:Ce^3+^ NSC and Ru PS was developed. Ru/radioPDT showed higher singlet oxygen generation with moderately increased potential in PC3 cell killing efficiency over PPIX/radioPDT. Favorable characteristics of size, surface charge, stability, and cellular uptake were maintained. Together, this shows the suitability of Ru/radioPDT NPs in improving efficiency for anticancer radiation therapy in pre-clinical and eventual clinical scenarios.

## Data availability statement

The raw data supporting the conclusions of this article will be made available by the authors, without undue reservation.

## Ethics statement

Ethical approval was not required for the studies on humans in accordance with the local legislation and institutional requirements because only commercially available established cell lines were used.

## Author contributions

All authors listed have made a substantial, direct, and intellectual contribution to the work, and approved it for publication.
