# Supplementary material for: High quantum efficiency ruthenium coordination complex photosensitizer for improved radiation-activated Photodynamic Therapy
Source: Front Oncol. 2023 Aug 28;13:1244709. doi: 10.3389/fonc.2023.1244709 (PMC10494715; doi:10.3389/fonc.2023.1244709)
Supplement: Supplementary file 1 [file DataSheet_1.docx]

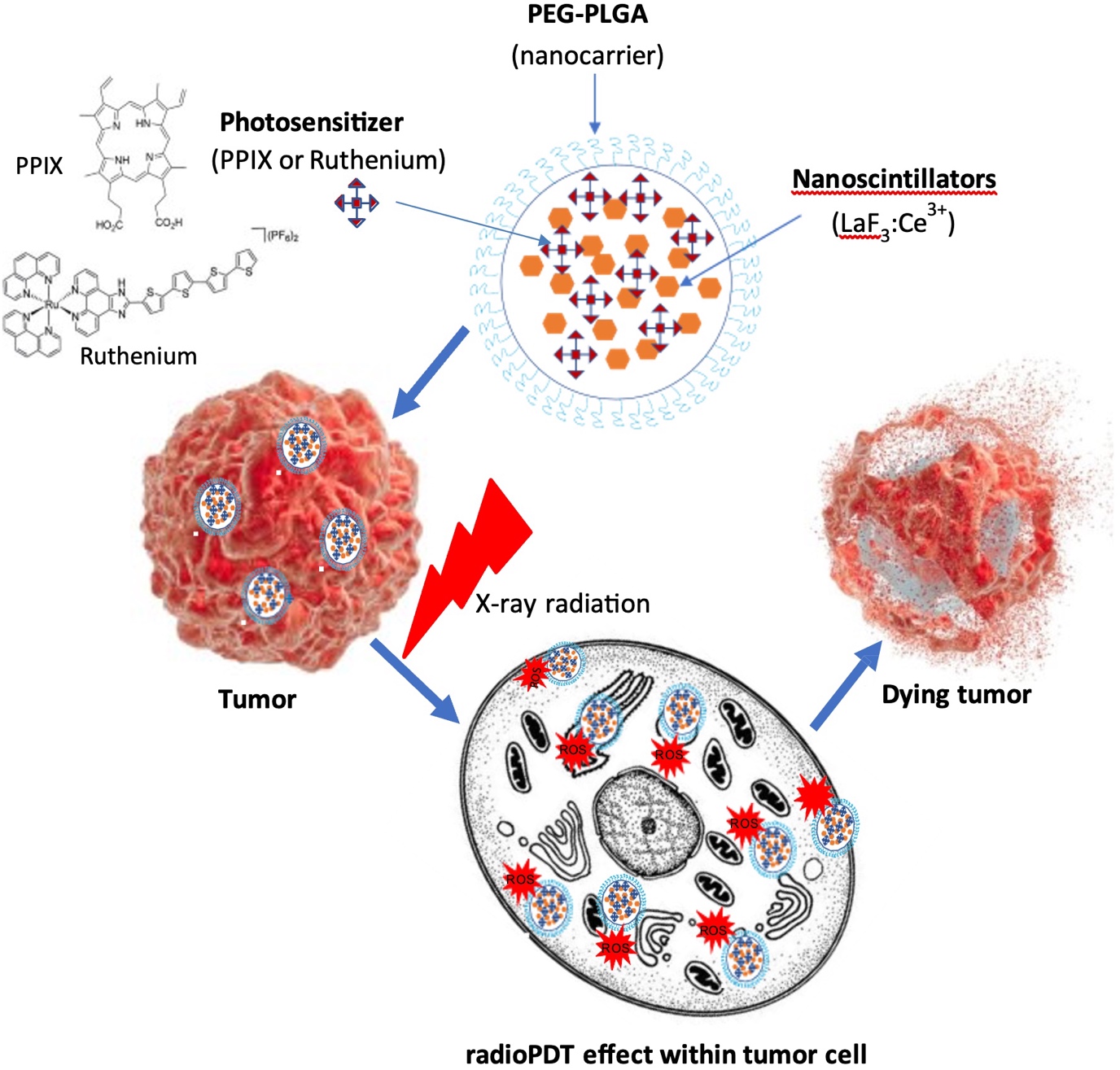


Figure S1. Schematic illustration of mechanism of tumor killing action of PEG-PLGA encapsulated nanoscintillators and photosensitizer (PPIX or Ruthenium) upon radiation activation.

**Supplemental flowchart 1: Synthesis of nanoscintillators (NSC)**

CeCl_3_ plus LaCl_3_ solution was added dropwise to NH_4_F solution

NH_4_F was kept in continuous stirring at 70 °C oil bath

Stirring for 2 hrs

Ultracentrifugation to collect the resultant nanoscintillators

Dissolved in water

CeCl_3_

LaCl_3_


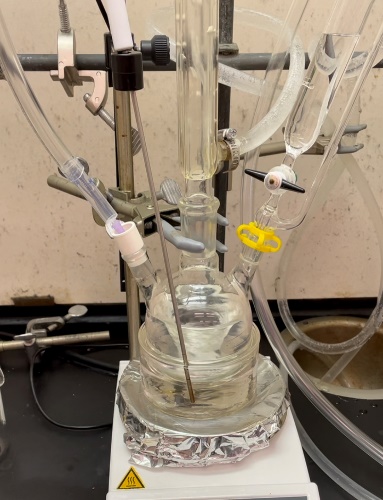


Oil bath at 70 °C

NH_4_F

**Supplemental flowchart 2: Loading of nanoscintillators (NSC) and photosensitizer into PEG-PLGA**

PEG-PLGA, NSC and photosensitizer were suspended in acetonitrile

Nanoprecipitation: Acetonitrile mixture was added drop-wise to 20X excess volume of milliQ water

Acetonitrile was removed by vacuum evaporation

Stirring for overnight

NPs were purified and concentrated using tangential flow filtration

**Supplemental Table S1: Zeta potential of radioPDT NPs**

|  | **0 h** | **48 hrs** |
| --- | --- | --- |
| PPIX/radioPDT | -27.4 ± 0.5 mV | -23.2 ± 1.1 mV |
| Ru/radioPDT | -17.4 ± 0.7 mV | -10.8 ± 1 mV |


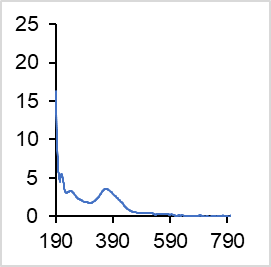

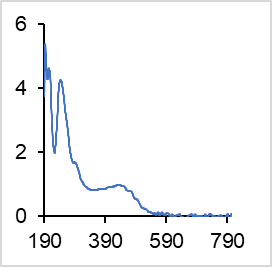

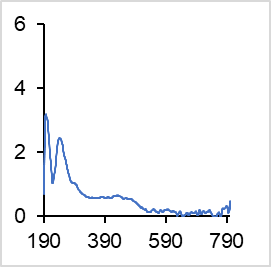

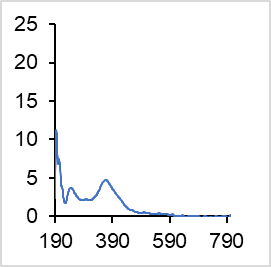

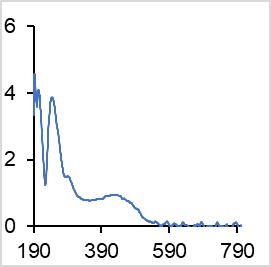

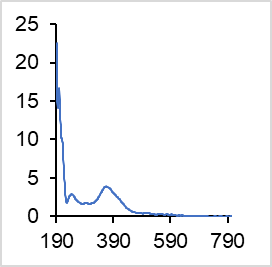


PPIX/radioPDT, 24 hrs

PPIX/radioPDT, 48 hrs

PPIX/radioPDT, 0 h

Ru/radioPDT, 0 h

Ru/radioPDT, 24 hrs

Ru/radioPDT, 48 hrs

Absorbance (a.u.)

Absorbance (a.u.)

A

B

PPIX/radioPDT, 0 h

PPIX/radioPDT, 48 hrs

Ru/radioPDT, 48 hrs

Absorbance (a.u.)

Absorbance (a.u.)

Supplemental Figure S2: UV-Vis absorption spectrum of PPIX/radioPDT (A) and Ru/radioPDT (B). Samples were incubated in 8% FBS containing DMEM for indicated time points.


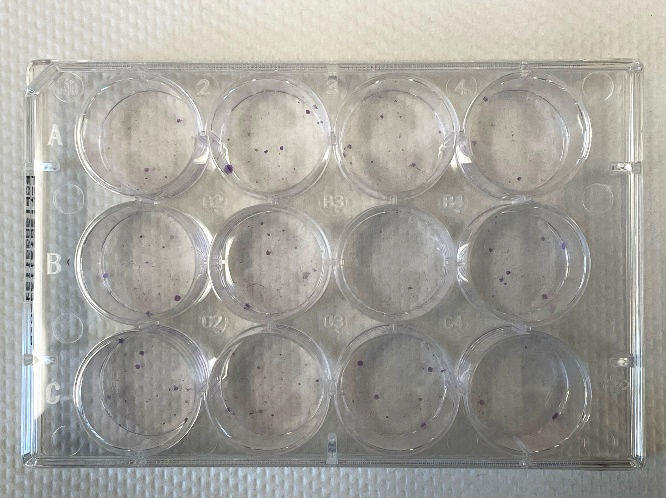

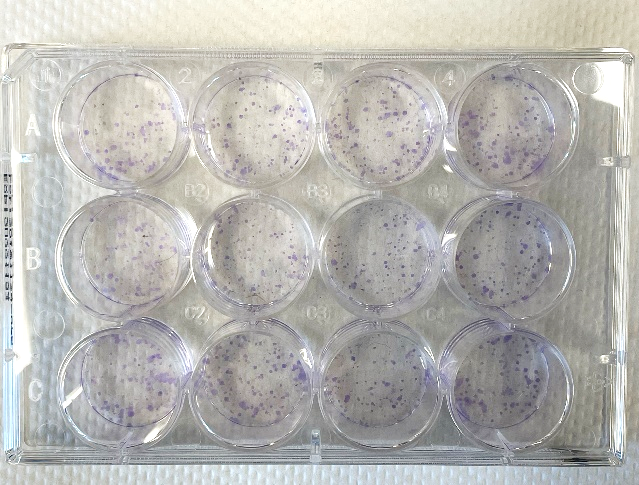

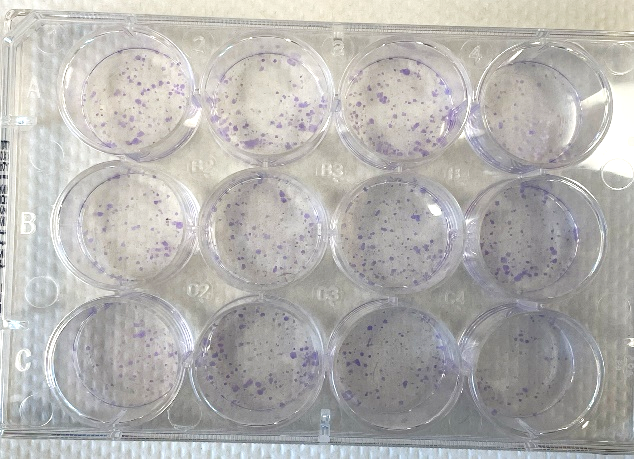

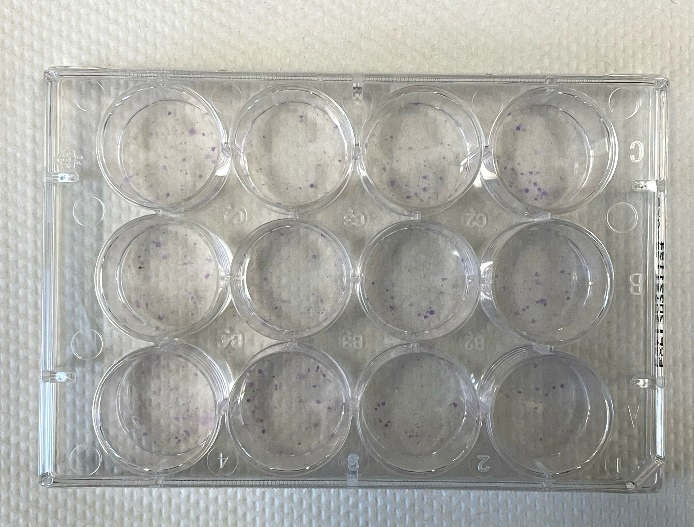


PEG-PLGA

NSC-NP

PPIX

Ru

PPIX/radioPDT

Ru/radioPDT

No radiation

3 Gy

Untreated

Untreated

Supplemental Figure S3: Colony forming ability of PC3 cells treated with radioPDT NPs and radiation. PC3 cells were treated with radioPDT NPs and an equivalent amount of control reagents and then irradiated with 3 Gy single dose radiation.
